# A cautionary note on the use of chromosome conformation capture in plants

**DOI:** 10.1186/s13007-017-0251-x

**Published:** 2017-11-16

**Authors:** Suraj Jamge, Maike Stam, Gerco C. Angenent, Richard G. H. Immink

**Affiliations:** 1 Laboratory of Molecular Biology, Wageningen University & Research, Droevendaalsesteeg 1, 6708 PB Wageningen, The Netherlands; 2 Wageningen Plant Research, Bioscience, Wageningen University & Research, Droevendaalsesteeg 1, 6708 PB Wageningen, The Netherlands; 3 Swammerdam Institute for Life Sciences, Universiteit van Amsterdam, Science Park 904, 1098 XH Amsterdam, The Netherlands

**Keywords:** 3C, *Arabidopsis*, Plants, Chromatin interactions

## Abstract

**Background:**

The chromosome conformation capture (3C) technique is a method to study chromatin interactions at specific genomic loci. Initially established for yeast the 3C technique has been adapted to plants in recent years in order to study chromatin interactions and their role in transcriptional gene regulation. As the plant scientific community continues to implement this technology, a discussion on critical controls, validations steps and interpretation of 3C data is essential to fully benefit from 3C in plants.

**Results:**

Here we assess the reliability and robustness of the 3C technique for the detection of chromatin interactions in *Arabidopsis*. As a case study, we applied this methodology to the genomic locus of a floral integrator gene *SUPPRESSOR OF OVEREXPRESSION OF CONSTANS1 (SOC1)*, and demonstrate the need of several controls and standard validation steps to allow a meaningful interpretation of 3C data. The intricacies of this promising but challenging technique are discussed in depth.

**Conclusions:**

The 3C technique offers an interesting opportunity to study chromatin interactions at a resolution infeasible by microscopy. However, for interpretation of 3C interaction data and identification of true interactions, 3C technology demands a stringent experimental setup and extreme caution.

**Electronic supplementary material:**

The online version of this article (10.1186/s13007-017-0251-x) contains supplementary material, which is available to authorized users.

## Background

Perception and response to internal and external stimuli is the fundamental nature of cellular life. The transcriptional regulatory system plays an integral role in fulfilling the needs of the cell and organism by ensuring proper gene activity. In comparison to bacteria, transcriptional control of an eukaryotic cell is far more complex, involving several layers of regulation inside the nucleus. It takes more than just the action and sufficient quantity of activator or repressor proteins to modulate gene expression.

Many modules, such as transcription factors (TFs), RNA polymerase, chromatin remodellers and associated proteins, and regulatory DNA sequences, are determinants of eukaryotic transcription [1, 2]. All together these factors create an open chromatin structure, which is essential to initiate eukaryotic gene transcription. General TFs recognize and bind to discrete DNA sequences (also referred to as *cis*-elements) located in the core promoter region close to the transcription start site (TSS). For instance, the TATA box is one such evolutionarily conserved core promoter *cis*-acting element found upstream of most eukaryotic genes [3–5]. Upon association of the general TFs to *cis*-elements, they interact with other proteins and form complexes to recruit RNA polymerase II, thereby initiating transcription. Examples of these other proteins include specific TFs, which can bind to *cis*-elements more distantly located from the core promoter elements [6, 7]. When these distant *cis*-elements are involved in the specific activation of gene expression they are called transcriptional enhancers. These enhancers can be found upstream, downstream, or within introns of coding regions and are reported to be located as far as several hundred kilo bases (kb) from the TSS [8, 9]. These distant enhancers can come into close proximity of their target sequences by protein-mediated chromatin interaction. In this respect, transcriptional gene regulation relies to a great extent on proteins that bind to DNA, not only close to the genes that they regulate, but also at distal DNA sites that can interact with the transcription initiation site by looping the intervening DNA. Thus DNA looping is speculated to be crucial to allow multiple proteins to regulate the core transcriptional machinery, resulting in a correct and controlled transcriptional response [10–12].

Over the last two decades much attention has been paid to the role of chromatin and its conformation in the regulation of gene expression. Various processes, including the differential deposition of histone variants, histone modifications such as methylation and acetylation, DNA methylation, and the activity of other non-histone architectural proteins are known to regulate the structure of chromatin [13–15]. Empirical evidence add to the notion that the dynamics of higher-order chromatin conformation plays a crucial role not just in transcription, but also in other nuclear processes inherent to DNA (DNA replication, DNA repair, chromosome transmission etc.). Therefore understanding the conformation of the chromatin within the cell nucleus has become a fundamental topic in biology.

Over the years, different imaging methods have been deployed to study chromosome conformation [16, 17]. However, detailed and local analysis of chromatin contacts with these methods has been complicated due to technical constraints. For instance, scanning electron microscopy (SEM) provides high resolution, but this technology is laborious, and most importantly, not suitable to study specific loci. Light microscopy has a limited resolution (200 nm), and therefore is inadequate to define local chromosome conformation. Direct in vitro evidence of DNA looping has been shown using very-high resolution three-dimensional atomic force microscopy (AFM) [18]. Nevertheless, this method is labour intensive and an in vitro based approach. Artificial TFs fused with fluorescent proteins such as GFP do allow to spatially visualize and temporally track repetitive genome sequences in vivo, but the method still needs optimization in order to visualize unique individual loci and to detect chromatin interactions [19]. Fluorescence in situ hybridisation (FISH) is another alternative. However, this method involves stringent preparation treatments that can influence the chromatin organization itself and it was originally only suitable for the visualization of repetitive sequences [20]. Though, recent improvements and coupling of FISH with rolling-circle amplification of gene-specific circularizable oligonucleotides makes it possible to visualize the dynamics of individual loci [21]. Overall, microscopy studies have been crucial in defining chromosome territories and nuclear architecture at a single-cell level and new developments will probably allow to image individual chromatin contacts in vivo in the near future. Additionally, a new molecular approach has become available in recent years to study spatial organization of chromosomes at a high resolution, and this molecular tool is called **C**hromosome **C**onformation **C**apture (3C) [22].

In 3C, chromatin in the intact nucleus is cross-linked by formaldehyde, followed by digestion with a restriction enzyme (RE) and intramolecular ligation [22]. The 3D conformation of the region or locus of interest is then studied by detecting ligation events occurring between non-neighbouring restriction sites. Possible interactions occurring between different chromosomal locations within the nucleus can be quantified as fused sequences by quantitative PCR [23]. The 3C method is cell population based and results in information about the relative frequency of interactions. The 3C method was initially developed for yeast by Dekker and co-workers and has been widely adapted to different model organisms shortly after. For plants, this method was also successfully applied to study chromatin conformation [24] and, since then it has become a powerful method to study gene looping in plants, as summarized below.

The first report exploring 3C in plants comes from a study in maize (*Zea mays*) that describes the role of a distant enhancer sequence at the *b1* locus. At the *b1* Locus, a hepta-repeat around 100 kb upstream of the transcription start site (TSS) appeared to interact with the TSS region in a tissue and epiallele-specific manner [8]. Since then a number of studies have highlighted the occurrence of chromosomal interactions in Thale Cress, *Arabidopsis thaliana*.

Crevillen et al. reported the presence and condition-dependent disruption of a chromatin loop at the *FLOWERING LOCUS C* (*FLC*) locus upon vernalization [25]. *FLC,* a potent floral repressor and a polycomb target gene, is under tight control of winter cold. Vernalization is a classical epigenetic process in which prolonged cold exposure quantitatively affects the time of flowering. A robust gene loop, due to an interaction between the 5′ and 3′ flanking sequences of the *FLC* locus, has been reported and this interaction is independent of the level of *FLC* transcript in different genetic backgrounds and genomic contexts. However, upon vernalization, within the first 2 weeks of cold exposure, the loop is disrupted and it has been proposed that this disruption is an early event in the transition of the *FLC* locus to an epigenetically silent stage. Subsequently, other DNA contacts in the *FLC* locus increase in frequency under control of the COLDAIR and COLDWRAP long non-coding RNAs (LncRNAs), giving rise to polycomb-dependent and stable repression of *FLC* expression [26].

Another study by Liu et al. in 2013 reported the occurrence of a conformational change in chromosome looping at the *TERMINAL FLOWER1* (*TFL1*) locus that appears to be regulating *TFL1* transcription [27]. In this case, disruption of the gene loop between the TSS and 3′ distal region of the *TFL1* locus results in *TFL1* suppression.

Likewise, two independent studies have identified gene loops at another flowering related gene, *FLOWERING LOCUS T* (*FT*), that are associated with the photoperiod-dependent flowering response [28, 29]. *FT,* a floral integrator, can unite signals from multiple pathways to induce flowering. The first study reports the occurrence of multiple loops between a distal enhancer element (that contains CCAAT boxes) and core *cis* regulatory sites located in the promoter of *FT (pFT*) [28]. Nuclear Factor-Y (NF-Y) is known to bind *CCAAT*-boxes and these CCAAT-bound NF-Y complexes are hypothesised to come into close proximity with core *pFT* sites, enabling improved recruitment and stabilized binding of CONSTANS (CO), together initiating photoperiod-dependent flowering in *Arabidopsis* [28]. A second study showed the folding of the *FT* locus into a three-dimensional structure, favouring interactions between two regulatory regions (named as *Block A* and *Block C,* ~ 5.6 kb apart) with another region called *Block ID,* an intermittent promoter region between *Block C* and *A* [29]. An introduced change in the promoter length of *FT*, i.e., an increase in the distance between *Block C* and *Block ID,* by a T-DNA insertion, abolished the C-ID interaction and resulted in reduced chromatin interactions of Block C with Block A. It is noteworthy to mention that the chromatin interactions identified in these two studies do not overlap.

Together, the 3C studies discussed above provide intriguing insights into the possible roles of chromatin interactions to regulate gene expression in plants, similar to the studies reported in yeast and other model organisms [30, 31].

3C provides an interesting opportunity to study in vivo chromatin interactions at a high-resolution and thus has become a standard method for studying chromatin contacts at single gene loci [8, 24]. However, like every other method, 3C has its own shortcomings. The challenges and technical issues of this method can at times outweigh its advantages. Therefore a good experimental setup, rigorous controls, and unbiased data analysis are crucial for meaningful interpretation of 3C data. This is clearly evident from studies performed in other model organism (mammalians, yeast etc.), where several papers have highlighted the importance of necessary 3C controls and appropriate data analysis [23, 32–35]. However, cautionary notes are largely missing in the plant science community. In this study we assessed the reliability and robustness of the qPCR-based 3C method in *Arabidopsis*. Based on this investigation, we provide detailed guidelines on necessary controls and how interaction data should be interpreted in a 3C experiment. Intricacies of this promising but challenging technique are further discussed.

## Results and discussion

### Chromosome conformation capture (3C) in *Arabidopsis*

**Fig. 1:**
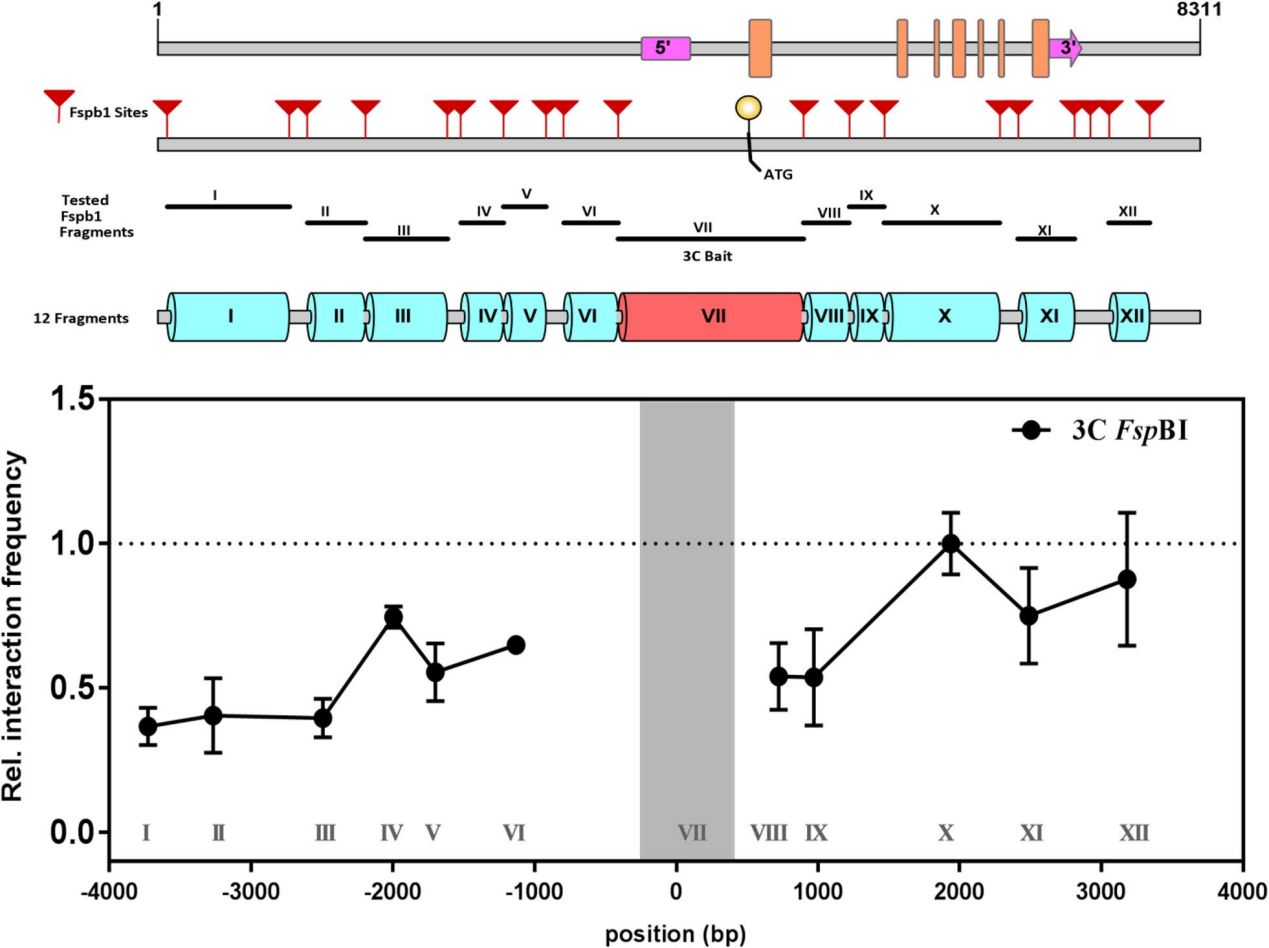
Chromosome conformation capture at the *SOC1* locus of *Arabidopsis* using the *Fsp*BI restriction enzyme. In the top panel a schematic diagram of the *SOC1* locus is shown indicating the positions of all *Fsp*BI restriction sites (red arrowheads), and the fragments tested for interaction with a region spanning the Transcriptional Start Site (TSS; Fragment VII). The pink blocks indicate the 5′ and 3′UTR and the orange blocks highlight the position of exons along the *SOC1* locus. The graph shows the observed relative interaction frequency of regions with the anchor VII, performed on 3 week-old wild type (Col-0) seedlings. Relative interaction frequencies are plotted on the y-axis. Distances in base pairs (bp) relative to the TSS of *SOC1* are plotted along the x-axis. Mean (± SD) derived from three independent biological samples is indicated. High interaction frequencies with the bait fragment indicate potential chromatin interactions

To assess the reliability and robustness of the 3C technique for the detection of chromatin interactions in *Arabidopsis*, we used this methodology to investigate the chromatin conformation at the locus of the floral integrator gene *SUPPRESSOR OF OVEREXPRESSION OF CONSTANS1 (SOC1)* [36]. Initially, the *SOC1* locus, including the ~ 3.8 kb promoter, the gene body, and ~ 1 kb downstream region, was divided into fragments using the four-cutter RE *Fsp*BI, as schematically shown in Fig. 1. Twelve distinct fragments of varying lengths (smallest fragment IX of 276 bp and longest fragment VII of 1475 bp), spanning the entire *SOC1* locus, were tested for chromatin contacts. Fragment VII, which contains the transcriptional start site (TSS), was used as the bait (also referred to as 3C anchor) to generate a chromatin interaction profile (Fig. 1). Throughout this study, proper controls were used as described previously [24] (also see “Methods” section) to ensure that only valid chromatin contacts are detected and quantified. As seen in Fig. 1, multiple contacts between the anchor and other regions of the *SOC1* locus were identified. Overall the 3C interaction profiles observed were consistent and reproducible across independent biological samples.

In a 3C experiment the fragment(s) that show(s) the highest interaction frequency with the bait fragment is (are) considered as chromatin contact(s). For the TSS region in the *SOC1* locus the highest interaction frequencies were observed with fragments X, XI and XII, all downstream of the VII-bait (Fig. 1). Furthermore, a potential contact with a promoter region, Fragment IV, was identified.

### Cross-validation of chromatin contacts

**Fig. 2:**
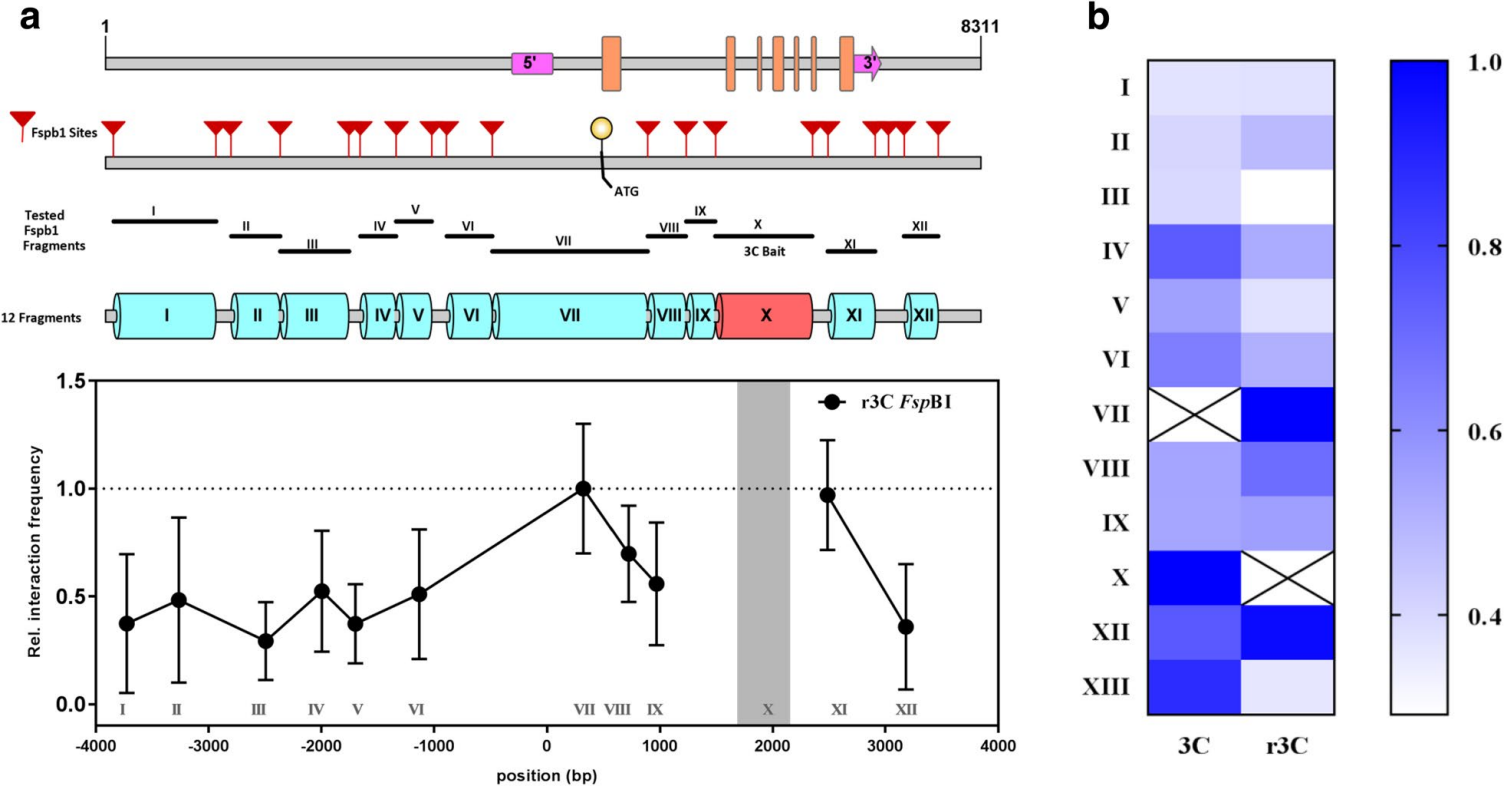
Reciprocal chromosome conformation capture (r3C) at the *SOC1* locus of *Arabidopsis* using the *Fsp*BI restriction enzyme. **a** Schematic diagram of *SOC1* locus showing the position of all *Fsp*BI restriction sites, and the fragments tested for interaction. The pink blocks indicate the 5′ and 3′UTR and the orange blocks highlight the position of exons along the *SOC1* locus. The graph shows the outcome of the r3C analysis of *SOC1* with *Fsp*BI using fragment X as bait. The experiments were performed on the same 3C libraries used in the original 3C experiment presented in Fig. 1. Mean (± SD) derived from three biological replicates is plotted. **b** A heat map summarizing and comparing the 3C and r3C interaction profile obtained with *Fsp*BI. The cross indicates the fragment used as bait. Note that the highest reciprocal contact frequency was identified between fragment VII and X. The intensity of blue colouring is an indication of the relatively interaction frequency (scale from 0-1.0)

One way to validate the putative chromatin contacts identified from a 3C experiment, is by performing a reciprocal 3C (r3C) experiment. In an r3C experiment, the fragment showing the highest interaction frequency with the 3C bait in the initial assay is used as a new 3C bait to generate an interaction profile. Thus we performed r3C experiments using one of the potential interacting fragments downstream of the *SOC1* TSS as bait (Fragment X; Fig. 1) aiming to verify the identified contacts. The chromatin interaction profile generated using fragment X as bait is shown in Fig. 2. As expected, we identified a high interaction frequency for the combination X-VII, validating the initial identified 3C contact point (Fig. 1). Moreover, another region (XI) showed an equally high interaction frequency when X was used as bait. However, since XI is in immediate proximity of the 3C bait, this high interaction frequency might be the result of random collisions of neighbouring fragments, a phenomenon often observed in 3C experiments [32].

The key difference between the 3C and r3C experiment is the fragment that is selected as bait and consequently, the combinations of qPCR primers used to detect the relative interaction frequency. For the *SOC1* locus we identified in this way e.g., an interaction between bait VII and fragment X (Fig. 1) and therefore fragment X was used as bait in the r3C experiment (Fig. 2). In any PCR-based 3C experiment, the primer of the bait is kept constant and is combined with a unique primer annealing specifically to one of the fragments that is tested for interaction [8]. It is good to realize that in a 3C and r3C experiment the combination of primers to test the interactions between one specific combination of fragments (VII and X in the example of *SOC1*), is identical. Performing an r3C experiment is certainly of value, since a comprehensive profile of chromatin interaction of the locus will be obtained from yet another viewpoint. However, it is good to realize that the outcome of 3C experiments might be biased due to different characteristics of the used REs or technical constraints of PCR [37]. Therefore, it is desirable to perform another independent type of validation, besides the r3C experiment.

### Validation of 3C interaction profiles with another restriction enzyme

**Fig. 3:**
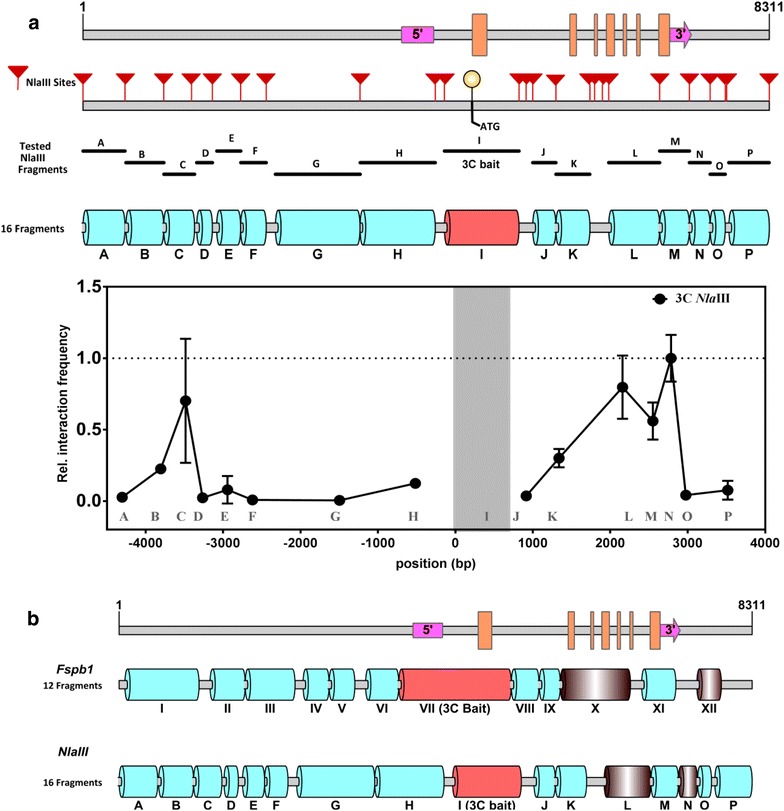
Chromosome conformation capture at the *SOC1* locus of *Arabidopsis* using the *Nla*III restriction enzyme. **a** Schematic diagram of *SOC1* locus showing the positions of all *Nla*III restriction sites (red arrowheads), and the fragments tested for interaction with a region spanning the Transcriptional Start Site (TSS; Fragment I). The pink blocks indicate the 5′ and 3′UTR and the orange blocks highlight the position of exons along the *SOC1* locus. The graph represents the results of the 3C analysis on 3-week old wild-type (Col-0) seedlings using fragment I as 3C bait. Mean (± SD) for two biological replicates plotted. **b** A schematic representation of the top two interacting fragments (in brown) identified at the *SOC1* locus with *Fsp*BI and *Nla*III

One of the best options to confirm and validate the outcome of a 3C experiment is to repeat the 3C experiment with yet another RE. A similar 3C interaction profile obtained from two independent REs strengthens the reliability of identified chromatin contacts. Further, it allows a more precise identification of the specific chromosomal regions that interact. With this in mind, we re-examined the chromatin interaction profile for the *SOC1* locus using a different four-cutter enzyme (*Nla*III). Now 16 distinct fragments of varying length spanning the entire *SOC1* locus were tested for chromatin contacts (Fig. 3). The interaction profile of this validation 3C assay is shown in Fig. 3a. Fragment I spanning the TSS, which to a large part overlaps with Fragment VII used as bait upon the *Fsp*BI digestion (Fig. 1), was used as bait. The highest interaction frequency was observed for the combination ‘I–N’. In addition to that, bait I also interacted with fragments L and C at a relatively high frequency. By comparing the 3C profiles obtained with the two REs (Fig. 3b), we identified at least one contact to overlap in both 3C experiments with the region spanning the TSS, and this is represented by the regions X and L. However, region N was found as novel interacting region for bait I, but this region was not represented in the *Fsp*BI run due to multiple closely located *Fsp*BI restriction sites. Consequently, this part of the locus became too fragmented for reliable qPCR primer design and amplification and was not monitored in the *Fsp*BI-based experiment. Besides this lack of coverage of some regions due to the selected RE, tested fragments do not completely overlap and this can result in differences. In the case of *SOC1 for example*, it is possible that the interaction between bait VII and region XII detected in the *Fsp*BI experiment is due to a contact between a sequence in the 5′-end of bait VII, and therefore not identified in the *Nla*III experiment fragment I (Fig. 3b).

**Fig. 4:**
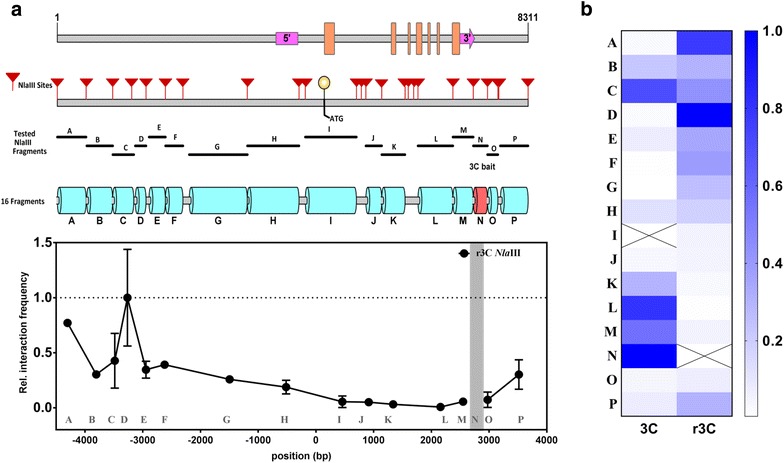
Reciprocal chromosome conformation capture (r3C) at the *SOC1* locus of *Arabidopsis* using the *Nla*III restriction enzyme. **a** Schematic diagram of the *SOC1* locus showing the position of all *Nla*III restriction sites (red arrowheads), and the fragments tested for interaction. The pink blocks indicate the 5′ and 3′UTR and the orange blocks highlight the position of exons along the *SOC1* locus. The graph represents the results of the r3C analysis on 3-week old wild type (Col-0) seedlings with fragment N as bait. Mean (± SD) for two biological replicates is plotted. **b** A heat map summarizing and comparing the 3C and r3C interaction profile performed with *Nla*III. The cell with a cross indicates the bait. The intensity of blue colouring is an indication of the relatively interaction frequency (scale from 0-1.0)

Although not all potential interactions were validated, the results obtained with the second RE supports the initially identified interaction between a fragment around the *SOC1* TSS and a fragment towards the end of the coding region of the gene (X for *Fsp*BI and L for *Nla*III, respectively). The next logical step was to perform an r3C experiment using *Nla*III. For this purpose fragment N, located in the 3′-region of *SOC1* (Figs. 3 and 4a) was used as a bait, as this region showed the highest interaction frequency with region I (Fig. 3a). Surprisingly, the observed relative interaction frequency for the combination N–I in the r3C experiments was extremely low, suggesting no interaction (Fig. 4). Instead we identified two other potential contact points, both located in the *SOC1* promoter, i.e., fragments A and D. Notably, we obtained a similar deviating result in all biological replica’s that were tested. Although we cannot exclude the possibility that this result is due to the way interaction frequencies are calculated and interpreted, the outcome (Fig. 4) shows the importance of applying multiple validation and confirmation experiments, such as r3C and the use of a second RE.

**Fig. 5:**
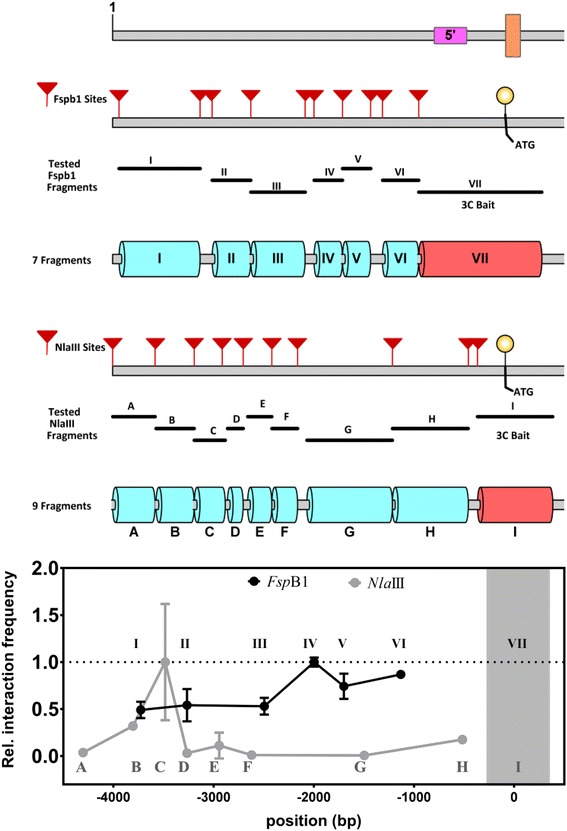
Chromosome Confirmation Capture at the *SOC1* promoter only. In the top panel a schematic diagram of *SOC1* promoter is given, showing the position of all *Fsp*BI and *Nla*III restriction sites, along with the fragments monitored for interaction. The graph represents the results of the 3C analysis based on the *FspB*I and *Nla*III data sets and using fragment VII and I as bait, respectively

The observed contradicting results prompted us to investigate potential reasons of miss-interpretation of 3C results due to the lack of sufficient controls or technical constrains of the 3C technology. A user of 3C defines e.g., the bait region and the size of the region of interest to study, and hence the number of potentially interacting fragments to be monitored. These choices affect the 3C outcome, since the measured interactions are relative to one another with the highest interaction frequency set as one. In literature, we commonly come across 3C studies focusing on promoter regions only, the entire gene locus, or a specific distal enhancer region to identify e.g., promoter-enhancer contacts. When we re-analyzed our data starting from the hypothesis that there is an interaction between the TSS and an upstream *SOC1* promoter region, and therefore monitored this part of the locus only, we observed the highest interaction between bait VII and promoter fragment IV with the *Fsp*BI restriction profile (Fig. 5). Although this chromosome interaction was also detected in our initial experiment (Fig. 1), our attention was directly drawn towards the region in the 3′-end of the *SOC1* gene, for which the highest relative interaction frequencies were found using bait VII. However, more worrying is the non-overlapping pattern at the *SOC1* promoter observed for the *Nla*III restriction profile, in which bait I interacts with promoter fragment C (Figs. 3a, 5). Surprisingly, this interaction pattern is not overlapping at all with the *Fsp*BI relative interaction pattern. Once more, it is possible that this deviation is caused by the lack of full overlap between the bait fragments. Sequences in the 3′-end of fragment I might be essential for the interaction with fragment C. Nevertheless, these results show how much the outcome depends on the choice of the RE to be used and which chromosomal region is taken and by that, the inclusion or exclusion of particular high interacting regions.

Overall, upon performing 3C at the *SOC1* locus independently with two different REs, we found various similarities, but also differences in the generated 3C profiles. One clear trend observed was the interaction of the TSS-spanning bait region with a region close to the 3′ UTR of the *SOC1* locus for both restriction profiles. A striking discrepancy were the interaction patterns identified for the TSS bait fragment with the promoter region. Hence, our data show the potential subjectivity of 3C results and prompt for cautiousness when interpreting 3C interaction patterns.

### Classification of 3C interactions

All the above discussed results reveal the subjectivity of 3C data interpretation. Furthermore, in a 3C experiment, for regions in close proximity to the bait fragment under study, usually a high occurrence of ligations due to random (non-specific) collisions is observed [32, 38]. Thus, mere identification of ligation events does not necessarily mean the occurrence of an existing in vivo interaction. In order to be able to detect interactions above the background of random interactions, it’s important to carefully consider the proximity between the bait and the supposed interacting fragments. The *Arabidopsis* genome in nature is very small and compact [39], hence studying regulatory interactions at individual gene loci is complex, due to the small distances between neighbouring restriction fragments thereby resulting in high potential for random interactions [38]. In conclusion, the combination of compact genome of *Arabidopsis* and the flexible nature of chromatin fibres makes interpretation of 3C interaction on individual loci challenging and demands high caution.

Reviewing published literature we found only a limited number of 3C studies that have been performed in plants. Among these studies we observed considerable shortcomings within the 3C experimental set up (e.g., the PCR method, lack of endogenous normalization and random ligation libraries as controls, use of only one enzyme and no r3C), and differences in the way the 3C interaction data have been interpreted and represented. Furthermore, comparing 3C results across experiments and laboratories is complicated due to differences in the experimental set up, such as PCR method, normalization method, plant growth conditions, and the way the interaction frequency is determined. For example, in literature, one can find chromatin interactions determined using 3C by either semi-quantitative PCR or by qPCR-based approaches. Only the latter provide numeric peak interactions and is therefore a much more trusted and widely adopted method in recent years. A recent publication manually curated more than 3000 interactions from 5000 publications across 17 species into a database called 3C database (3CDB) [40]. This 3CDB classified the strongest 3C interactions into four distinct classes based on their reliability. Class I and II cover the semi-quantitative PCR data, which nowadays is not an accepted detection method, whereas Class III and IV refer to numeric peak interactions. According to the 3CDB classification, interactions belonging to class IV are considered to be the most reliable, due to the fact that they are validated with an r3C experiment [40]. The results we described in this study are all numeric peak interactions and fall into class III and IV. However, based on our interaction profiles from independent 3C experiments that used different REs, we see a need to further extend this set of classifications. We suggest introducing a class V for interactions that have been identified and r3C-validated using two different REs. Taking into account that even in this set up the experimental procedure is the same and provides relative and hence, subjective data, 3C experiments should be confirmed by an alternative and independent method to get full proof for a potential chromosome interaction.

### Challenges of the 3C method

Ligation based methods, such a 3C, heavily rely on a sound experimental design. Many technical biases may be introduced if the design and set up of the experiment is not optimal. For example, optimization of crosslinking conditions is necessary, as over-fixed chromatin often renders digestion with REs inefficient. Similarly, biases may arises from the choice of RE and its digestion efficiency. One of the prerequisites for REs in 3C is their ability to digest crossed-linked chromatin efficiently, but at the same time providing the desired resolution at the locus of interest. By far not all REs behave optimally in 3C, e.g., because buffering conditions during digestion are sub-optimal (e.g., due to presence of detergents). Hence, optimization of several parameters is essential in order to overcome technical biases in a PCR-based 3C experiment. Most of these technical aspects and their optimization procedures have been very-well addressed in literature [23, 24, 32, 41–43]. Besides these technical issues, biological variation may arise from differences in the growing conditions, the time of tissue collection in relation to the developmental age of the plant, and due to sampling itself. These aspects are very critical, especially when the goal is to study dynamics in chromatin interactions in time or upon a change in condition, as was e.g., done for the *FLC* locus [25]. To exemplify this, we performed a 3C assay on a developmentally different tissue where *SOC1* is known to be actively expressed [44]. For this experiment we sampled the same type of plant material exactly 1 week later, but upon transfer of the plants to flowering-inducing long day (LD) conditions and after growth at these conditions for 7 days (7dai). At this time point, we observed a distinct chromatin interaction profile at *SOC1* locus, when using the *Nla*III RE (Additional file 1: Fig. S1), suggesting dynamics in relation to *SOC1’s* transcriptional state. However, when 3C was repeated on this sample using *Fsp*BI (Additional file 1: Fig. S2), we obtained a pattern resembling the pattern after 3 weeks growth under SD conditions (Fig. 4b), suggesting lack of dynamics. This example reveals that extra caution is required when studying dynamic interactions and that it is of utmost importance to keep biological variation at minimum.

### Beyond the traditional 3C

Since the development of the 3C method, many variants of this technology have been rolled out (reviewed in [45, 46], Table 1). These variants enabled the user among others to study chromatin interaction at a genome-wide scale. 4C combines the traditional 3C assay with microarrays (3C-on-chip) [47], and thereby a user can examine one-to-all contacts throughout the genome, instead of exploring one-to-one locus specific contacts as is done in classical 3C experiments. Advancements and development of cost-effective sequencing techniques gave birth to a wide range of sequencing variants of the 3C method (Table 1), improving the overall resolution of the interaction profile. Instead of using one viewpoint, some 3C variants, such as multiplex 3C-seq (many-to-all) and Hi-C (all-to-all), provide the opportunity to explore genome-wide interactions from multiple viewpoints simultaneously. More and more computational tools and packages are now publically available making it easier to process and analyse the vast amount of genome-wide interaction data [48–53]. Thus in comparisons to traditional 3C, nowadays some 3C variants might be more attractive, robust, and cost effective to perform. Therefore, we recommend users to compare the ins-and-outs of all these methodologies taking into account their research question, before deciding on the appropriate 3C method of choice (reviewed in [46]). However note that the full potential of all these variants still remains to be exploited in plants. A few studies did make use of 4C and Hi-C approaches to gain insight into the three-dimensional chromatin configuration of *Arabidopsis* genomes [54–58]. In comparison to the majority of other plant genomes, the *Arabidopsis* genome is densely packed with a gene density of one gene per 4.5 kb. However, most of the chromosome conformation capture technologies are best suited to study mid-range and long-range chromatin interaction and therefore, less suitable for *Arabidopsis*. Nevertheless, one Hi-C study did report contact maps of up to 2 kb resolution [57]. But, when it comes to studying short-range interactions, all the above discussed variants are limited in resolution in comparison to the (q)PCR-based 3C methods. Further improvements in sequencing depths, choice of REs (e.g., micrococcal nuclease, four cutters) and overcoming computational barriers may drastically improve the resolution of these technologies in the near future, enabling the generation of unbiased high-resolution chromatin interaction maps.

**Table 1:** Overview of existing and recently developed 3C-based methods

Ligation based chromatin capture method	Application	References
qPCR-based 3C	One-to-one	[23, 24]
3C-seq, 4C	One-to-all	[59, 60]
5C	Many-to-many	[61]
ChIA-PET	Many-to-many	[62]
Multiplex 3C-seq	Many-to-all	[63, 64]
HiCap,CHi-C	Many-to-all	[65–67]
Capture-C	Many-to-all	[68]
T2C	Many-to-all	[69]
Hi-C, Dnase Hi-C, Micro-C, Micro-CXL,	All-to-all	[67, 70–73]
TCC	All-to-all	[74]

## Conclusions

3C is a powerful tool when it comes to studying chromatin interactions at a gene specific locus. However, identification of valid interactions via PCR-based 3C demands multiple controls and validation steps. Only when the results are consistent across the proper control and validation experiments, an interaction can be considered of high confidence. Subsequently, it is of interest to unveil for every high confidence interaction whether it exists because of the regulation of gene expression, a particular nuclear or chromatin organisation, or spatial restrictions in the nucleus or the flexible nature of chromatin. Hence, identified interactions do not reveal the underlying mechanism behind its co-localization, neither do they distinguishing if it’s a functional or non-functional interaction. To shed more light on the functionality of an observed interaction, genetic studies are essential. For instance, making use of T-DNA insertion lines, or targeted disruption of the DNA regions involved in the observed interactions with CRISPR-Cas9 genome editing [75], can aid further functional characterization of identified in vivo interactions.

Since the establishment of the 3C technique, hundreds of potential interactions have been reported supporting the potential role of chromatin interactions in transcriptional control. As the plant scientific field is gaining momentum in deciphering this new layer of transcriptional regulation of intricate gene regulatory networks, the 3C technique will play a prominent role in expanding our knowledge on this new fundamental topic of plant biology. Nevertheless, utmost care should be taken in assigning meaningful 3C interactions, as described here.

## Methods

### Plant material, growth conditions and tissue collection

Col-0 wild type plants were grown on rock-wool for 3 weeks at 20 °C under short day (SD) conditions (8 h light, 16 h dark). Two grams of seedling material (above ground tissues) per biological sample were collected during the afternoon hour of the day. In addition, material was sampled 7 days later and after transfer and growth of the plants for a week at long day photoperiod conditions (16 h light, 8 h dark).

### Chromatin conformation capture (3C)

3C was performed on the *SOC1* locus using the previously described protocol with some adaptations for *Arabidopsis* [24]. Two grams of *Arabidopsis* above-ground seedling material was crosslinked with 2% paraformaldehyde PBS buffer under vacuum for 30 min (mins) on ice. The cross-linking reaction was stopped by addition of ice-cold 2 M glycine (final concentration = 0.125 M) under vacuum for 5 min on ice. The crosslinked tissue was ground and nuclei were isolated and purified using nuclei extraction (NE) buffer. Before digestion, the purified nuclei pellets were re-suspended in 1.2 × restriction buffer and treated with 0.2% SDS at 65 °C for 20 min. Later, SDS was sequestered by incubating with 2%Triton X-100 for 30 min. 3C analysis was performed on the *SOC1* locus using two different REs (namely *Fsp*BI and *Nla*III) independently. 400 U of RE was used for overnight digestion at 37 °C. Digestion was stopped by incubation at 65 °C for 20 min. Ligation was performed using 100 U of T4 DNA ligase, initially at 16 °C for 5 h, followed by room temperature for 45 min. Reverse cross-linking was done overnight with a treatment of proteinase K at 65 °C. After reversal of the crosslinks, phenol/chloroform extraction and ethanol precipitation was performed for recovery of the DNA.

### 3C primers, controls and quantification

All the primers used in this study are listed in Tables 2 and 3. For a detailed discussion on controls we highly recommend these published studies [32, 43]. The relative interaction frequencies of one fragment to another were calculated based on quantitative PCR (qPCR) data using SYBR Green I master mix. For the analysis of the specific ligation events, two controls were used. First, in order to correct for the primer amplification efficiencies, for each primer pair the qPCR dataset was normalized with an random ligation (RL) control sample. The RL control sample was obtained by digestion of a BAC clone containing the *SOC1* locus and followed by re-ligation in small volumes to obtain all the possible random ligation events. Secondly, in order to control for the quality and quantity of each 3C sample, the 3C data needed to be further normalized to 3C values measured for an endogenous control locus (usually a reference gene) unrelated to the *SOC1* locus. The chromatin state of such a reference gene is assumed to be stable across samples. Therefore, for each 3C sample, 3C values were also obtained for the reference gene *TIP41*-*like*. The reference gene primer amplification efficiencies were also corrected with a RL control obtained by digestion and re-ligation of a BAC clone containing the *TIP41*-*like* locus. The 3C data of *SOC1* was normalized to the 3C values measured for the *TIP41*-*like* locus to obtain relative interaction frequencies. For more details on step-by-step data analysis of the qPCR-based 3C method see [76]. All figures shown in this study are the mean of two or three independent biological samples.

**Table 2:** 3C primers for *Fsp*BI restriction profile

Name	Gene locus and restriction enzyme	Primer on	Seq 5′ to 3′
PDS6848	SOC1_*Fsp*BI	Fragment I	AGATTCTCAAACATCAGTCGGA
PDS6849	SOC1_*Fsp*BI	Fragment II	ACAAAAGGAGTAGGTTTCTGGA
PDS6850	SOC1_*Fsp*BI	Fragment III	TGAGCTTATGACTGGTAAACTC
PDS6851	SOC1_*Fsp*BI	Fragment IV	GTTTTGGATTTGTCTCAACCAG
PDS7489	SOC1_*Fsp*BI	Fragment V	TGGTCCTCCTCCCGATATAGA
PDS6852	SOC1_*Fsp*BI	Fragment VI	ACGAGAGAGTGTTTGTGTCC
PDS6847	SOC1_*Fsp*BI	Fragment VII (Bait)	GACGTTTGCTTTGAGAGGTG
PDS6853	SOC1_*Fsp*BI	Fragment VIII	GCTTCATTTCATGCTCATTCC
PDS6854	SOC1_*Fsp*BI	Fragment IX	ACTTCTTTCTCTCGAACCTACT
PDS6855	SOC1_*Fsp*BI	Fragment X	AGTAAGTAAGCCTCTTGTGCT
PDS6856	SOC1_*Fsp*BI	Fragment XI	AGCTGCTTCTCTCTTGTTGT
PDS6857	SOC1_*Fsp*BI	Fragment XII	AAGGGCCTACTTTGCGATAA
PDS7307	TIP41_Like *Fsp*BI	Bait-TSS	GTTTCGATCTCCCAGTCATG
PDS7308	TIP41_Like *Fsp*BI	− 500 bp	AACTAAACCAAAGCAAATACGA

**Table 3:** 3C primers for *Nla*III restriction profile

Name	Gene locus and restriction enzyme	Primer on	Seq 5′ to 3′
PDS7922	SOC1_*Nla*III	Fragment A	ACCGTTGGATGAAAGAGCAT
PDS7923	SOC1_*Nla*III	Fragment B	CGCGTCTACAGAAAGTTAACCA
PDS7924	SOC1_*Nla*III	Fragment C	TGACCTTACCCACATAGAAACAC
PDS7925	SOC1_*Nla*III	Fragment D	GCCAAACCAACATCACAAAA
PDS7926	SOC1_*Nla*III	Fragment E	GAAAACAAAAGGAGCGAAAAA
PDS7927	SOC1_*Nla*III	Fragment F	TTTTTCCCACCCTTATTTCTC
PDS7928	SOC1_*Nla*III	Fragment G	CATTGCCCCATTGTCTCTGT
PDS7920	SOC1_*Nla*III	Fragment H	ATCCTCGAAAGCTTCCTCCT
PDS7929	SOC1_*Nla*III	Fragment I	AATCATCTGTCTCTCTCTTTCTCAA
PDS7930	SOC1_*Nla*III	Fragment J	TGAAAATGCCAGCTTTTGAT
PDS7931	SOC1_*Nla*III	Fragment K	GAGCGGTAATGAATATAACCACAA
PDS7932	SOC1_*Nla*III	Fragment L	TTGGTTATCTTCAATCATCAACCT
PDS7933	SOC1_*Nla*III	Fragment M	TGATTCTGAACTGCTTGTGTTATG
PDS7934	SOC1_*Nla*III	Fragment N	ATCCATTGGCCAAAAATCAA
PDS7935	SOC1_*Nla*III	Fragment O	GAGGCTTTTAGCCCATCAAA
PDS7936	SOC1_*Nla*III	Fragment P	CGACGTCGCACGATTTATTA
PDS7939	TIP41_Like *Nla*III	Bait-TSS	CCGGCCTAGTTTCATTTTAGTT
PDS7940	TIP41_Like *Nla*III	− 1000	CGAGCACAAATACAAAACCG

## Additional file

**Additional file 1.** **Fig. S1** 3C analysis at the *SOC1* locus 7 days after the transfer of short day grown plants to long day flowering inducing conditions (7dai) on *Arabidopsis* rosette tissue using the *Nla*III restriction enzyme. **Fig. S2** 3C analysis at the *SOC1* locus 7 days after the transfer of short day grown plants to long day flowering inducing conditions (7dai) on *Arabidopsis* rosette tissue using the *Fsp*BI restriction enzyme.
