# Supplementary figures and images for: A cautionary note on the use of chromosome conformation capture in plants
Source: Plant Methods. 2017 Nov 16;13:101. doi: 10.1186/s13007-017-0251-x (PMC5691870; doi:10.1186/s13007-017-0251-x)

## Slide 1
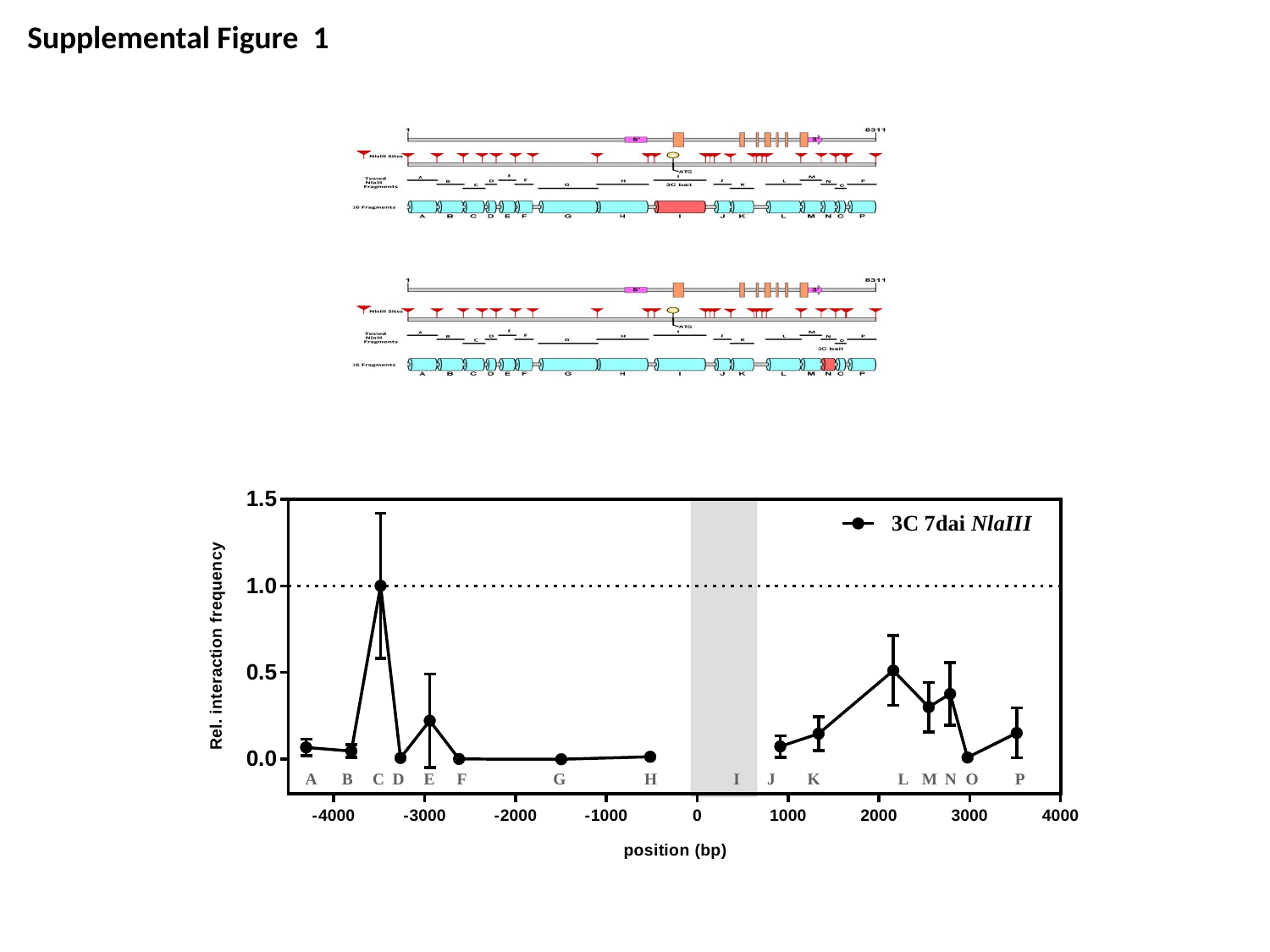

Supplemental Figure 1

## Slide 2
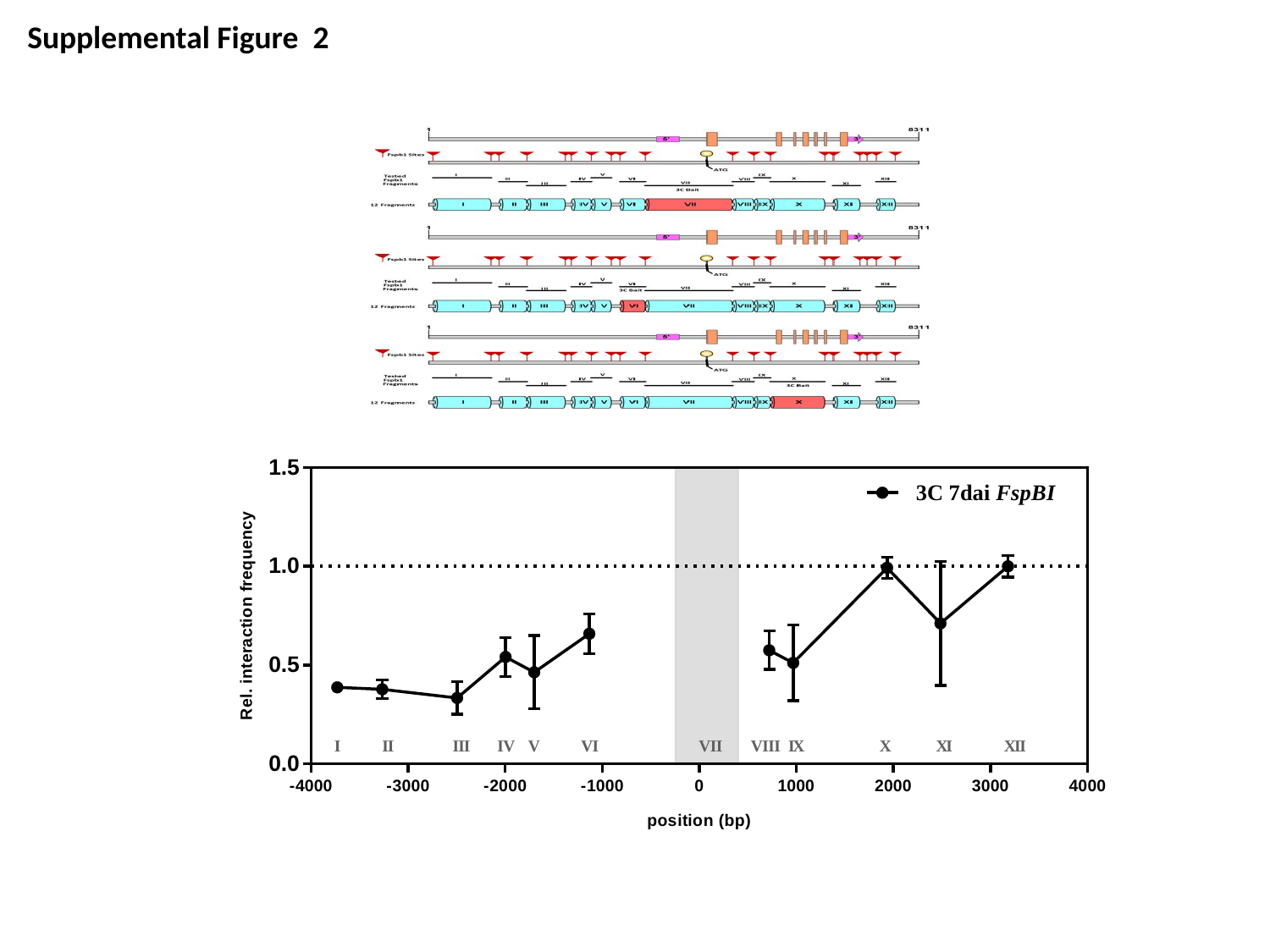

Supplemental Figure 2

Supplement: Supplementary file 1 — Additional file 1. Fig. S1 3C analysis at the SOC1 locus 7 days after the transfer of short day grown plants to long day flowering inducing conditions (7dai) on Arabidopsis rosette tissue using the NlaIII restriction enzyme. Fig. S2 3C analysis at the SOC1 locus 7 days after the transfer of short day grown plants to long day flowering inducing conditions (7dai) on Arabidopsis rosette tissue using the FspBI restriction enzyme. [file 13007_2017_251_MOESM1_ESM.pptx]
